# Effects of Environmental Conditions on the Fitness Penalty in Herbicide Resistant *Brachypodium hybridum*

**DOI:** 10.3389/fpls.2017.00094

**Published:** 2017-02-03

**Authors:** Eyal Frenkel, Maor Matzrafi, Baruch Rubin, Zvi Peleg

**Affiliations:** The Robert H. Smith Institute of Plant Sciences and Genetics in Agriculture, The Robert H. Smith Faculty of Agriculture, Food and Environment, Hebrew University of JerusalemRehovot, Israel

**Keywords:** fitness cost, herbicide resistance, photosystem II inhibitors, *psb*A gene, seed bank, weed control

## Abstract

Herbicide-resistance mutations may impose a fitness penalty in herbicide-free environments. Moreover, the fitness penalty associated with herbicide resistance is not a stable parameter and can be influenced by ecological factors. Here, we used two *Brachypodium hybridum* accessions collected from the same planted forest, sensitive (S) and target-site resistance (TSR) to photosystem II (PSII) inhibitors, to study the effect of agro-ecological parameters on fitness penalty. Both accessions were collected in the same habitat, thus, we can assume that the genetic variance between them is relatively low. This allow us to focus on the effect of PSII TSR on plant fitness. S plants grains were significantly larger than those of the TSR plants and this was associated with a higher rate of germination. Under low radiation, the TSR plants showed a significant fitness penalty relative to S plants. S plants exhibiting dominance when both types of plants were grown together in a low-light environment. In contrast to previous documented studies, under high-light environment our TSR accession didn’t show any significant difference in fitness compared to the S accession. Nitrogen deficiency had significant effect on the R compared to the S accession and was demonstrated in significant yield reduction. TSR plants also expressed a high fitness penalty, relative to the S plants, when grown in competition with wheat plants. Two evolutionary scenarios can be suggested to explain the coexistence of both TSR and S plants in the same habitat. The application of PSII inhibitors may have created selective pressure toward TSR dominancy; termination of herbicide application gave an ecological advantage to S plants, creating changes in the composition of the seed bank. Alternatively, the high radiation intensities found in the Mediterranean-like climate may reduce the fitness penalty associated with TSR. Our results may suggest that by integrating non-herbicidal approaches into weed-management programs, we can reduce the agricultural costs associated with herbicide resistance.

## Introduction

Since plants were first domesticated ∼10,000 years ago, crop plants have been exposed to recurrent infestations by weed plants. To date, weeds are the most important biotic factor affecting agriculture production and causing yield losses in various crops worldwide [e.g., *Corylus avellana* (Kaya-Altop et al., 2016), *Zea mays* (Soltani et al., 2016) and *Oryza sativa* (Chauhan and Johnson, 2011; Chauhan and Opena, 2012)]. Chemical control using herbicides is considered the most cost-effective and efficient method of weed management. However, the use of routine herbicide applications to reduce weed infestations also imposes continuous selective pressure on diverse weed populations and can lead to the evolution of herbicide resistance (Jasieniuk et al., 1996; Kaundun et al., 2012; Busi and Powles, 2013). Herbicide resistance had been reported in more than 240 weed species, including resistance to almost every known herbicidal mode of action (MOA; Heap, 2016).

Photosystem II (PSII) inhibitors include several herbicide chemistries (e.g., triazine, triazinone, and substituted urea). Herbicides that work via this MOA compete with the plastoquinone B (PQ_*B*_) at the PQ_*B*_ binding site located on the D_1_ protein of the PSII complex enzyme (Arntzen et al., 1982), causing the formation of free radicals, which lead to plant death (Fuerst and Norman, 1991). Due to their effectiveness, PSII inhibitors are routinely used for weed control in agro-systems, forests, and roadsides. The first reported case of resistance to a PSII inhibitor in 1970 involved resistance to simazine in common groundsel (*Senecio vulgaris*; Ryan, 1970). Since then, resistance to PSII inhibitors has become widespread all over the world; 231 cases of resistance have been reported for atrazine alone (Heap, 2016). Mechanisms of resistance to PSII inhibitors in weeds typically involve an altered target site (TS; Heap, 2014). Several different point mutations in the *psb*A gene have been showed to confer TSR to PSII inhibitors (e.g., Hirschberg and McIntosh, 1983; Mengistu et al., 2005; Park and Mallory-Smith, 2006; Mechant et al., 2008; Perez-Jones et al., 2009; Thiel and Varrelmann, 2014). TS resistance is related to chloroplastic gene (*psb*A), which lead to maternally inheritance resistance (e.g., Plowman et al., 1999). Non-target site resistance to PSII inhibitors is less abundant, but there are some cases that have been reported over the years. Both chlorotoluron and isoproturon resistance was found to be related to enhanced metabolic activity [e.g., *Alopecurus myosuroides* (Hyde et al., 1996) and *Phalaris minor* (Singh et al., 1998), respectively].

Plants’ adaptation to environmental conditions is characterized by the selection of a natural population toward a phenotype that best suits the prevailing environmental conditions (Fisher, 1930). Moreover, alleles that confer higher adaptive value in one environment may have a detrimental impact on fitness in another environment (Orr, 2005). Fitness penalty under herbicide-free environments as result of gerbicide-resistance mutations was reported for several MOA’s such as acetyl-CoA carboxylase (Vila-Aiub et al., 2005), 5-enolpyruvylshikimate-3-phosphate synthase (Yanniccari et al., 2016) and photosystem II (Benyamini et al., 1991; Matzrafi et al., 2014) inhibitors. This can be associated with the fact that it alters the natural function of important biological processes in the cell (Ahrens and Stoller, 1983; Vermaas and Arntzen, 1983). The fitness penalty associated with resistance to PSII inhibitors is not a fixed parameter and its magnitude is influenced by ecological factors such as radiation (Holt and Radosevich, 1983), temperature (Vencill et al., 1987), inter-accession competition (Conard and Radosevich, 1979) and inter-species competition (Williams et al., 1995). It has also been suggested that fitness penalties may be more evident under stressful environmental conditions (Vila-Aiub et al., 2009).

The fitness penalty associated with TSR to PSII inhibitors has been reported to involve different physiological and biochemical aspects, such as significantly reduced photosynthetic potential (Holt et al., 1981; Ahrens and Stoller, 1983), reduced vegetative growth (e.g., Holt, 1988), delayed flowering (e.g., Beversdorf et al., 1988), reduced reproductive potential (e.g., Weaver and Warwick, 1982), decreased competitive ability (e.g., Conard and Radosevich, 1979) and more damage from photo-inhibition (e.g., Sundby et al., 1993). The magnitude of the fitness penalty associated with resistance to PSII inhibitors implies that in an herbicide-free environment, there will be strong selective pressure against mutations in the *psb*A gene. This subject had been modeled (Gressel and Segel, 1990) and validated in several different studies (Benyamini et al., 1991; Sibony and Rubin, 2003b).

*Brachypodium*, a Mediterranean temperate winter wild grass, has emerged as an attractive experimental model species for biotic and abiotic stress (Fursova et al., 2012; Tripathi et al., 2012; Benavente et al., 2013; Matzrafi et al., 2014; Shaar-Moshe et al., 2015). Recently, we identified a *Brachypodium hybridum* population in a planted forest that includes both individuals that are sensitive (S) to PSII inhibitors and individuals that exhibit TSR to those herbicides (Matzrafi et al., 2014). Here, we used two accessions, each exhibiting one of these phenotypes, to study the effect of ecological parameters on the fitness penalty associated with this TSR. By using accessions from the same habitat, we were able to minimize the genetic variation within the experiment (Cousens et al., 1997) and emphasize the fitness penalty associated with PSII TSR.

## Materials and Methods

### Plant Material and Growth Conditions

Sensitive (BrI-638, **S**) and resistant (BrI-637, **TSR**) *Brachypodium hybridum* accessions from the BrI Collection (Matzrafi et al., 2014) were used in this work. Both accessions were collected in the same habitat (planted forest), were atrazine was applied as a conventional practice to assist in the young trees establishment. Seeds from each accession were germinated in plastic trays (50 cm × 25 cm × 5 cm) filled with growth mixture (Tuff Marom Golan, Israel). The trays were placed in a dark, cold room (18°C) to break the seeds’ dormancy until germination. After emergence, young seedlings were transplanted as specified for each experiment below.

### Herbicide Dose Response

Seedlings of the S and R accessions were transplanted into 0.2-L pots (6 cm × 6 cm × 6 cm), one per pot, filled with growth mixture (Tuff Marom Golan, Israel) and grown in an environmentally controlled chamber (16/10°C, day/night). Thirty-day-old plants (3–4 leaves) were exposed to increased rates (0, 1/8, 1/4, 1/2, 1, 2, 4, 8, 16, 32 and 64 kg ha^-1^) of atrazine (Atranex^®^ 50% SC, ADAMA-Agan, 50% active ingredient) -X = 1000 g ha^-1^, using a chain-driven sprayer delivering 300 L ha^-1^, with four replicates. Twenty-one days after treatment (DAT), all of the aboveground tissue was harvested, oven-dried (80°C, 48°h) and weighed to obtain shoot dry weight (DW) data. Relative values were calculated by dividing the DW of the treated shoots by that of the control shoots.

### Phenotypic Measurements

Seedlings of the S and R accessions were transplanted into 0.2-L pots filled with growth mixture (one per pot) and grown in a net house under approximately 90% radiation (100% = 1000–1100 μmol m^-2^ s^-1^), a day/night temperature of 20/9°C and short-day (10 h of light) conditions. For the analysis of chlorophyll content, the flag leaf (10 mg) was sampled 31 days after transplanting and immersed in 2 mL of N,N-dimethylformamide in the dark for 48 h at 4°C. The absorbance of the supernatant at 647 and 664 nm was measured using a spectrophotometer (ST-VS-723, LAB-KITS, Hong Kong) and chlorophyll *a* and *b* concentrations were calculated as described in Moran (1982). Plants were harvested 79 days after transplanting. At that point, all aboveground tissue was harvested, oven-dried (80°C, 48 h) and weighed to obtain shoot DW data.

### Grain Shape and Emergence Rate

Subsamples of the S and TSR accessions (50 seeds per subsample) were weighed using an analytic scale (ED124S, Sartorius Weighing Technology GmbH, Germany), to obtain grain weight (GW) data. Grains were individually scanned using a flatbed scanner (HP Scanjet G2710, HP, USA) and grain length (GL), width (Gwid) and area (GA) were measured using the SmartGrain software (Tanabata et al., 2012). Emergence rates were then analyzed by planting 10 uniform seeds of each accession (S and TSR) 1 cm deep in 0.4-L pots (12 cm × 7 cm × 4 cm) filled with growth mixture. Ten pots of each accession were placed in a dark, cold room (18°C). Seedling emergence was recorded daily over 14 days.

### Effects of Radiation and Competition on Plant Productivity

Seedlings of the S and TSR accessions were transplanted into 4.5-L pots (26 cm × 16 cm × 11 cm), one per pot, filled with a mixture of 80% field brown-red degrading sandy soil (Rhodoxeralf; 76% sand, 8% silt and 16% clay) and 20% growth mixture at high density level that mimics non-agricultural natural conditions (480 plants/m^2^, 20 plants per pot). The pots were placed in a phytotron under short-day conditions (10 h of light) and temperatures of 16/10°C (day/night) for 48 days and then subjected to long-day conditions (14 h of light) at 22/16°C, to mimic the natural *Brachypodium* growing conditions in the Mediterranean climate. Experiments were organized in a two-factorial completely randomized design with four replicates for each treatment. Different radiation levels; 100% (control) and 40% (low), were used in this experiment. The low radiation level was achieved using a black shading net. Competition ability was examined either intra-accession (by transplanting plants from each accession separately) or inter-accession (by growing them in a mixture in the same pot). Plants were harvested 113 days after transplanting and tillers and spikes number were counted. All aboveground biomass was harvested and oven-dried (80°C for 48 h) and shoot DW was determined.

### Effect of Nitrogen Conditions on Plant Productivity

S and TSR seedlings were hydroponically grown in 15-L plastic tanks (35 cm × 29 cm × 15 cm) containing nutrient solutions (**Supplementary Table S1**) with two levels of nitrogen: 100% (control) and 6% (The lowest level that can be accurately measured in the system). The hydroponic solutions were continuously aerated and replaced every 7 days. Ten replicates of each treatment, with 16 plants in each plastic tank, were placed in a climate-controlled greenhouse (22/15°C day/night) under short-day conditions (10 h of light), in a completely randomized design. The plants were harvested at 56 days after transplanting, tillers and spikes were counted and all aboveground biomass was harvested, oven-dried (80°C for 48 h) and weighed to obtain shoot DW data.

### Effect of Competition with Wheat Plants on *B. hybridum* Productivity

S and TSR *B. hybridum* seedlings and bread wheat (*Triticum aestivum*, cv. Zahir) seedlings were transplanted into 15-L boxes (35 cm × 30 cm × 15 cm) filled with a mixture of 80% soil and 20% growth mixture. Each *B. hybridum* accession was arranged either alone (intra-accession competition) or together with wheat with equal number of plants (inter-species competition). A total of 28 plants were grown per box (to mimic normal field density of 266 plants/m^2^) in four replicates for each treatment, when both species were placed in the same box they were transplanted in a mixture. Plants were grown in a climate-controlled greenhouse (22/15°C day/night) under long-day (14 h of light) conditions, in a completely randomized design. At 60 days after transplanting, the numbers of tillers and spikes were recorded and all aboveground biomass was harvested, oven-dried (80°C for 48 h) and then weighed.

### Statistical Analyses

JMP Pro ver. 12 software (SAS Institute Inc., Cary, NC, USA), was used for all statistical analyses. Differences between two treatments were examined using Student’s *t*-test at a significance level of *P* ≤ 0.05. Analysis of variance (ANOVA) was performed to examine the effect of each single variable and interaction term. Dose-response curves were constructed by plotting the shoot DW data (21 DAT) for the different accessions as a percentage of that of the untreated control. These data were analyzed using SigmaPlot (ver. 10) software (Systat Software Inc., San Jose, CA, USA) and ED_50_ (herbicide rate reducing shoot FW by 50%) values were extracted. A non-linear curve model (sigmoidal logistic, three parameters; Seefeldt et al., 1995) was adjusted to analyze the effects of the tested herbicides in the different experiments.

$$Y=\frac{a}{1+(\frac{x}{X_{0}})b}$$

In the model, if *b* > 0, then *a* describes the upper limit of *Y. X*_0_ = ED_50_ and *b* describes the slope of the curve in ED_50_. The resistance index (RI) was calculated as the ratio of the ED_50_ value of the resistant accession to the ED_50_ of the sensitive accession.

## Results

### Plant Fitness Following the Application of Atrazine

**FIGURE 1:**
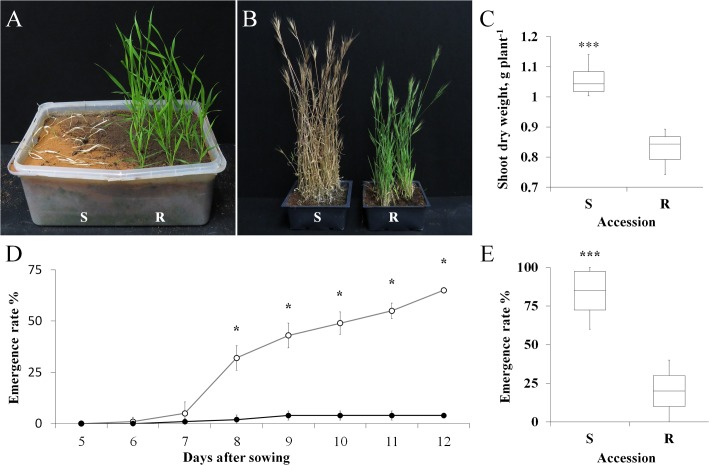
**(A)** A representative picture of sensitive (S, BrI-638) and resistant (TSR, BrI-637) *Brachypodium hybridum* accessions, 14 days after the application of atrazine (1 kg ha^-1^). **(B)** A representative picture of S and TSR plants grown in an herbicide-free environment, 130 days after transplanting. **(C)** Shoot dry weights of S and TSR accessions grown in an herbicide-free environment. **(D)** Time course showing the emergence rates of the S (open circles) and TSR (close circles) accessions. **(E)** Boxplot of the emergence rate of S and TSR accessions 60 days after sowing. Data are 1st, 2nd and 3rd quartiles and the minimum and maximum values among all of the data (*n* = 10). ^∗^ and ^∗∗∗^ indicate significant differences between accessions as determined by Student’s *t*-test at *P* ≤ 0.05 and *P* < 0.001, respectively.

*Brachypodium hybridum* accession BrI-637, previously shown to carry a mutation (A_790_→ G) in the chloroplast gene *psb*A (**TSR,** resistant), and BrI-638, which carries the WT gene (**S**, sensitive), (Matzrafi et al., 2014) were characterized for their response to PSII inhibitors. While the S accession was fully controlled by a 1/2X dose of atrazine, the TSR accession survived up to 64X of the recommended dose (**Figure 1A**; **Supplementary Table S2**; **Supplementary Figure S1**). Following herbicide application, the shoot DW of the R accession was significantly greater than that of the S accession, as assessed in terms of ED_50_ values (8.124 kg ha^-1^ vs. 0.161 kg ha^-1^, respectively).

### Plant Fitness under Natural Conditions in an Herbicide-Free Environment

The two *B. hybridum* accessions were grown in a nethouse under Mediterranean-winter conditions (i.e., vegetative growth under short-day conditions, followed by long-day conditions to induce flowering). The TSR accession began to flower 7 days later than the S accession (85 days vs. 92 days, respectively; **Figure 1B**; **Supplementary Table S3**). The shoot DW of the TSR accession was significantly lower than that of the S accession (0.82 g plant^-1^ vs. 1.05 g plant^-1^, respectively; **Figure 1C**). An analysis of flag-leaf chlorophyll content revealed significantly (*P* < 0.0001) higher chlorophyll *a* and *b* content in the S accession, as compared to the TSR accession (2.52 mg g^-1^ vs. 1.74 mg g^-1^ and 0.78 mg g^-1^ vs. 0.53 mg g^-1^, respectively; **Supplementary Table S4**). Notably, the chlorophyll *a/b* ratio of both accessions was similar (3.26 vs. 3.34, S and TSR accessions, respectively).

### Grain Shape and Emergence Rate

A time course of seed emergence revealed the significantly faster and greater emergence rate of the S accession, as compared with the TSR accession (**Figure 1D**). At the end of the experiment (14 days after transplanting), 84% of the S seeds had emerged, with only 20% emergence observed among the TSR seeds (**Figure 1E**).

**FIGURE 2:**
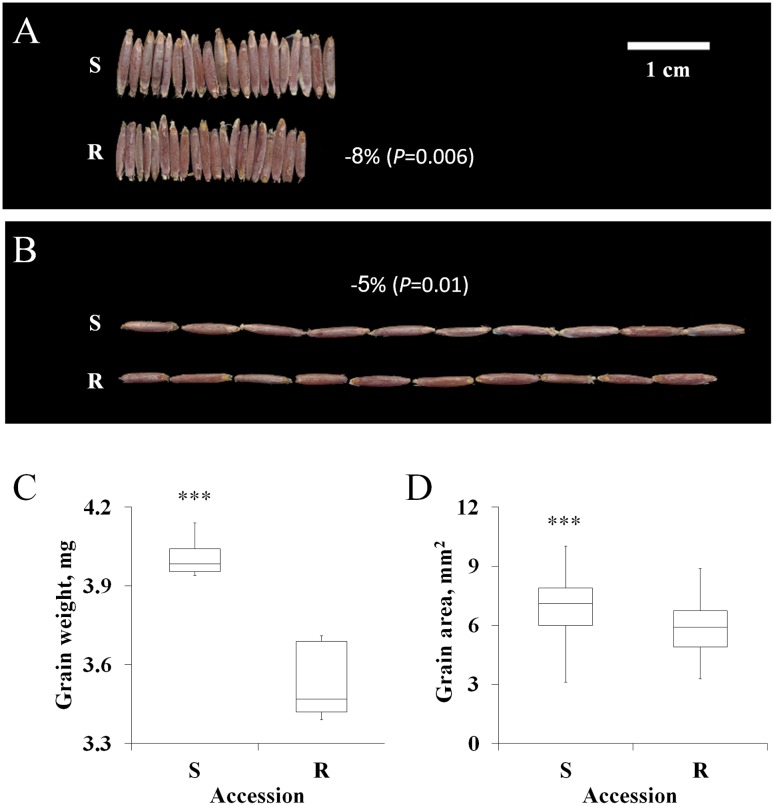
**Characteristics of the grain of the sensitive (S, BrI-638) and resistance (TSR, BrI-637) *Brachypodium hybridum* accessions.** **(A)** A representative picture showing grain width (*n* = 20) and **(B)** grain length (*n* = 10). **(C)** Boxplot of the grain weight and **(D)** grain area of the S and TSR accessions. Data are 1st, 2nd and 3rd quartiles and the minimum and maximum values among all of the data (*n* = 10 and 50, respectively). ^∗∗∗^ indicates significant differences, as determined by Student’s *t*-test at *P* < 0.001.

Previous studies involving various plant species have reported a positive correlation between fitness penalties and reproductive biomass (Ahrens and Stoller, 1983; Holt, 1988). In this study, the grains of the S accession were larger than those of the TSR accession (**Figures 2A–D**). S grains showed a significant advantage over the R grains in terms of total weight (4.01 mg vs. 3.53 mg), area (6.96 mm^2^ vs. 5.96 mm^2^), length (7.07 mm vs. 6.67 mm) and width (1.26 mm vs. 1.15 mm; **Figures 2C,D**; **Supplementary Table S5**).

### Effect of Environmental Conditions on the Ecological Fitness Penalty

Solar radiation is a key element in the plant’s photosynthetic productivity. When both accessions were grown in a controlled environment (in the phytotron) under natural level of light intensity (i.e., 100%), both accessions showed similar shoot DW (**Table 1**). When grown under low-light (i.e., 40%) conditions that mimic a cloudy environment, the TSR accession exhibited significantly (*P* = 0.015) less shoot DW, as compared with the S accession (0.56 g vs. 0.79 g, respectively; **Table 1**).

**Table 1:** Analysis of variance of the effect of *Brachypodium hybridum* accessions [sensitive (BrI-638) and resistant (BrI-637)], competition (intra- and inter-accession), and radiation level [high (100%) and low (40%)] on dry weight biomass production, number of tillers and number of spikes, under an herbicide-free environment.

Accession	Competition	Radiation	Dry weight, g	No. of tillers	No. of spikes
BrI-638	Intra-accession	High	1.06 ± 0.03	5.3 ± 0.21	5.8 ± 0.34
BrI-637	Intra-accession	High	0.91 ± 0.09	4.6 ± 0.48	5.0 ± 0.46
BrI-638	Intra-accession	Low	0.79 ± 0.03^∗^	3.8 ± 0.29	3.9 ± 0.28^∗^
BrI-637	Intra-accession	Low	0.56 ± 0.08	3.1 ± 0.21	3.5 ± 0.13
BrI-638	Inter-accession	High	1.35 ± 0.11^∗∗^	6.7 ± 0.36^∗∗∗^	6.9 ± 0.34^∗∗^
BrI-637	Inter-accession	High	0.88 ± 0.05	4.8 ± 0.17	5.0 ± 0.14

**Source**	**d.f.^1^**		**Sum of square**		

Accession (A)	1		0.668^∗∗∗^	7.031^∗∗∗^	8.168^∗∗∗^
Competition (C)	1		0.088	0.587	1.918^∗^
Radiation (R)	1		0.890^∗∗∗^	30.681^∗∗∗^	26.584^∗∗∗^
A × C	1		0.078	1.253	0.834
A × R	1		0.004	1.125	0.459
C × R	1		0.005	0.681	0.945
A × C × R	1		0.031	0.347	0.543
Error	24		0.693	7.431	7.715
Total	31		2.457^∗∗∗^	49.135^∗∗∗^	47.166^∗∗∗^


*^1.^d.f., degrees of freedom. ^∗^, ^∗∗^, and ^∗∗∗^indicate significance levels of *P* ≤ 0.05, *P* < 0.01 and *P* < 0.001, respectively.*

Nitrogen deficiency has a crucial effect on photosynthetic capacity and carbon fixation in plants (Khamis et al., 1990). Significant differences in shoot DW were observed between S and TSR plants grown hydroponically in nitrogen-rich (100%) and nitrogen-poor (6%) solutions. In the presence of an adequate (100%) nitrogen supply, TSR plants developed less shoot DW than S plants. The same trend was observed under deficient nitrogen conditions (6%). Another parameter of plant productivity is the number of spikes produced by the plant. Relative to the adequate (100%) nitrogen concentration, it appears that nitrogen deficiency (6%) had a greater effect on the production of spikes among the R plants (2 vs. 0.6, 100% vs. 6%, respectively) than among the S plants (9.9 vs. 6.6, 100% vs. 6%, respectively) plants (**Table 2**).

**Table 2:** Analysis of variance of the effect of *Brachypodium hybridum* accession [sensitive (BrI-638) and resistant (BrI-637)] and level of nitrogen [high (100%) and low (6%)] on dry weight biomass production, number of tillers and number of spikes, in an herbicide-free environment.

Accession	Nitrogen	Dry weight, g	No. of tillers	No. of spikes
BrI-638	High	0.93 ± 0.03***	10.4 ± 0.54	9.9 ± 0.41***
BrI-637	High	0.58 ± 0.03	9.5 ± 0.53	2.0 ± 0.49
BrI-638	Low	0.71 ± 0.02***	8.0 ± 0.19	6.6 ± 0.53***
BrI-637	Low	0.46 ± 0.03	8.1 ± 0.35	0.6 ± 0.24

**Source**	**d.f.^1^**	**Sum of square**		

Accession (A)	1	0.774^∗∗∗^	1.074	400.973^∗∗∗^
Nitrogen (N)	1	0.252^∗∗∗^	31.112^∗∗∗^	44.338^∗∗∗^
A × N	1	0.017	1.843	7.871^∗^
Error	30	0.182	49.003	44.775
Total	33	1.355^∗∗∗^	85.882^∗∗∗^	537.882^∗∗∗^


*^1^d.f., degrees of freedom. ^∗∗∗^indicate significance differences at levels of *P* < 0.001.*

### Competition between Weed Species

**FIGURE 3:**
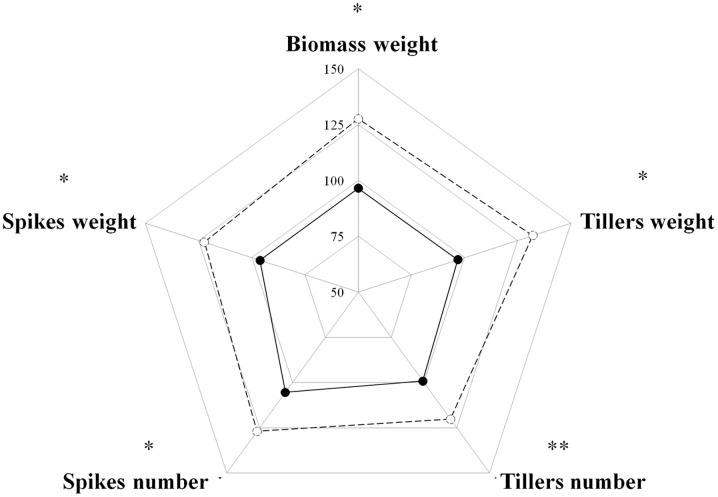
**Radar chart comparing the productivity parameters of the sensitive (S, BrI-638, dashed line) and resistant (TSR, BrI-637, full line) *Brachypodium hybridum* accessions (*n* = 4).** The data was analyzed using one-way ANOVA followed by Student’s *t*-test. ^∗^ and ^∗∗^ indicate significant differences between accessions; *P* ≤ 0.05 and *P* < 0.01, respectively.

Competition among plants of a single species can be divided into two types: intra-accession competition and inter-accession competition (Wiederholt and Stoltenberg, 1996). We examined both types of competition to gain a better understanding of the interaction between S and TSR plants under field conditions. Under intra-accession competition, plants from both S and TSR accessions had similar shoot DW levels (1.06 g vs. 0.91 g, respectively). In contrast, under inter-accession competition, TSR plants showed significantly less shoot DW than S plants (0.88 vs. 1.35, respectively; **Figure 3**; **Table 1**). Similar trends were noted for other parameters such as number of tillers and number of spikes (**Supplementary Table S4**).

### Competition between Wild Weeds and a Domesticated Crop

**FIGURE 4:**
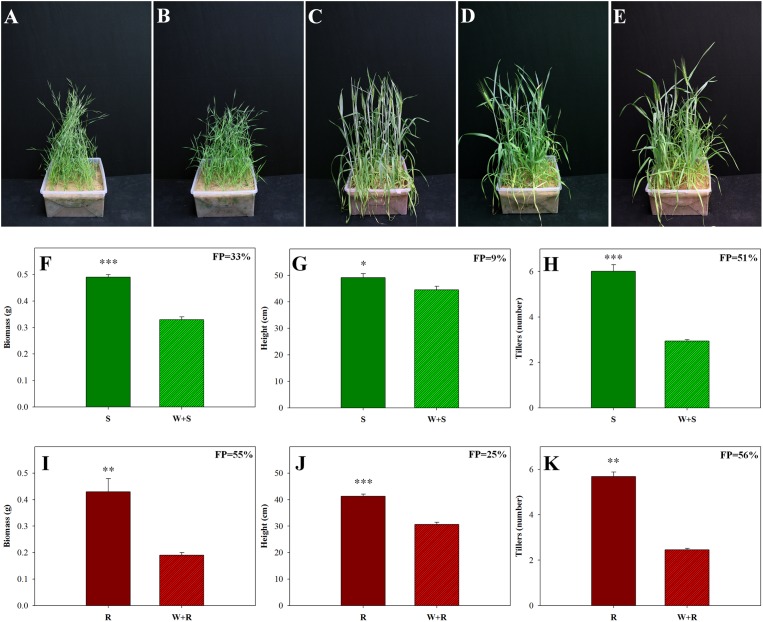
**Effect of intra- and inter-species competition between sensitive (S, BrI-638) and resistant (TSR, BrI-637) *Brachypodium hybridum* accessions and bread wheat (W, cv. Zahir) on plant phenology and productivity.** Representative pictures of **(A)** S plants, **(B)** TSR plants, **(C)** wheat plants, **(D)** competition between S and wheat plants (S + W) and **(E)** competition between TSR and wheat plants (R + W). Pictures were taken 60 days after transplanting. Comparison between S **(F–H)** and TSR **(I–K)** plants grown in competition with each other and in competition with wheat. **(F,I)** Biomass production, **(G,J)** plant height and **(H,K)** number of tillers. ^∗^, ^∗∗^, and ^∗∗∗^ indicate significant differences between accessions at *P* ≤ 0.05, *P* < 0.01, and *P* < 0.001, respectively.

Competition between two different species in the same habitat can significantly affect plant fitness. We investigated the competition of a wild weed (*B. hybridum*) with a domesticated cereal crop (*T. aestivum*) bred for uniformity and productivity (Borlaug, 1983). Comparisons of the S and TSR accessions grown in competition with wheat showed that all growth parameters of both accessions were affected by that competition (**Figure 4**). Examination of the agro-ecological performance of the S and TSR accessions revealed that the TSR plants were more negatively affected by competition than the S plants. Significantly less biomass (0.19 g vs. 0.33 g), shorter plants (30.7 cm vs. 44.6 cm) and fewer tillers (2.46 vs. 2.94) were observed among the TSR plants, as compared to the S plants, when each accession was grown in competition with wheat (**Figure 4**; **Supplementary Table S6**). The wheat plants showed similar levels of growth and productivity when grown in competition with the S and TSR weeds (**Figures 4C–E**; **Supplementary Table S6**).

## Discussion

Photosystem II inhibitors have been used as primary herbicides to control weeds in agro-ecological systems since the 1950s’. Over-use and misapplication have resulted in the evolution of increasing numbers of resistant weeds (Heap, 2016). Most cases of TSR to PSII inhibitors involves a single substitution of Gly_264_ (Heap, 2014). The fitness penalty caused by this mutation has been demonstrated in various resistant biotypes (Sundby et al., 1993; Arntz et al., 2000), as in the current study (**Figure 1B**). Reductions in photosynthetic efficiency (Ireland et al., 1988; Arntz et al., 2000), reproductive ability (Conard and Radosevich, 1979) and seed production (Park and Mallory-Smith, 2006) have all been reported to be correlated with PSII TSR.

Light intensity is a major factor limiting photosynthesis, affecting carbohydrate production and eventually growth (Mauseth, 2014). It has been suggested that under both low (Schonfeld et al., 1987) and high-light intensities (Hart and Stemler, 1990; Alfonso et al., 1996), the fitness of the mutated plants (TSR) is reduced relative to S plants. Accordingly, under lower radiation (400–450 μmol m^-2^ s^-1^), the S accession exhibited a significant advantage in biomass (74% vs. 61%, respectively; **Table 1**). The chlorophyll content of the R plants was less than that of the S plants, corresponding to the observed differences in biomass. However, in our high-radiation (∼1000 μmol m^-2^ s^-1^) treatment, there were no significant differences between the two accessions in terms of biomass. This experiment was repeated four times to validate these results.

Nitrogen is important for the production of chlorophyll and photosynthesis (Vos et al., 2005). While the S accession exhibited an advantage in terms of biomass production under control and low N conditions, the two accessions reacted similarly to the low-N treatment (82% of the control biomass vs. 79% of the control biomass for the S and TSR accessions, respectively; **Table 2**). These results suggest that the fitness penalty of the TSR accession can be associated with the efficiency of the photosynthetic apparatus (due to structural modification of the D1 protein), and less with chlorophyll density.

In natural environment, plants compete for resources such as water, light, and space. Vigorous plants that use resources more efficiently will eventually produce more seeds and dominate the population. When TSR and S accessions were grown together (inter-accession competition), the S biomass increased and the TSR biomass decreased, as compared to the biomass levels observed when the accessions were grown apart from one another (intra-accession competition; 1.35 g vs. 1.06 g and 0.88 g vs. 0.91 g, for the S and R, respectively; **Table 1**). Our results, as well as theoretical models (Gressel and Segel, 1990), indicate that in herbicide-free environments, the proportion of R plants in a population will decrease due to their low productivity in a highly competitive environment. Likewise, reductions of more than 30% in the reproductive ability of PSII TSR *Senecio vulgaris* and *Bromus tectorum* (Holt, 1988; Park and Mallory-Smith, 2005) were observed when those plants were grown in competition with S plants of the same species.

Following the examination of the competition between the TSR and S accessions, we also examined their relative fitness in a situation involving inter-species competition (*B. hybridum* / *T. aestivum*), to model an actual agricultural system. When these plants were grown in competition with wheat, reduced fitness penalties was observed for all of the examined growth parameters (biomass: -33% S vs. -55% TSR, height: -9% S vs. -25% TSR, number of tillers: -51% S vs. -56% TSR, respectively; **Figure 4**; **Supplementary Table S6**). Herbicide applications are usually carried out during the periods in which crop plants are most sensitive to the damage caused by weeds (Knezevic et al., 2002). In the current study, we observed a substantial reduction in the biomass and height of TSR plants under competitive conditions, which may indicate a lower competitive ability. By using non-herbicidal techniques such as crop competition, we can reduce the frequency of TSR seeds to a negligible level in the seed bank. These results are in accordance with those of other studies that have suggested the rapid extinction of TSR individuals under high field densities (reviewed by Vila-Aiub et al., 2015). One can assume that due to the high fitness penalty found in the current study, the TSR plants would be eliminated from this habitat. The presence of both TSR and S plants in the same habitat can be explained by two scenarios. The first scenario is as follows: The strong selection pressure of repeated PSII application resulted in a population shift that enriched the seed bank with R seeds, giving the R seeds/plants an advantage over the S seeds/plants (Rubin et al., 2004). The herbicide concentration required to control the R accession (BrI-637) was 50-fold higher than that needed to control the S accession (BrI-638; **Supplementary Figure S1**). As a consequence, under repeated herbicide applications, the TSR accession exhibited greater fitness (**Figure 1A**). Exclusion of these herbicides from the ecosystem (i.e., an herbicide-free environment created a shift toward S seed, due to the strong agro-ecological fitness penalty of the TSR accession. Due to seed bank enrichment with TSR seeds, this transition is still in progress and we are now witnessing the decline of TSR individuals in the population.

The second scenario is as follows: In Mediterranean environments, light intensities are very high (1000–1500 μmol m^-2^ s^-1^), compensating for the deficiency in the photosynthetic activity of TSR plants. These high levels of radiation can explain the abundance of TSR plants, due to the lower fitness penalty seen under high-light intensities. This can also explain the relative abundance of other PSII TSR mutant plants (e.g., *Conyza canadensis*; Matzrafi et al., 2015), as well as PSII TSR mutants of C_4_ plant species such as *Amaranthus retroflexus* (Van Oorschot and Van Leeuwen, 1984) and *Amaranthus blitoides* (Sibony and Rubin, 2003a), whose photosynthetic apparatus is more efficient than that of C_3_ plants.

## Conclusion

Both accessions examined in this study were collected from a planted forest in which multiple applications of PSII inhibitors were made in the past (to help the young trees establishment), but discontinued at least 20 years ago. The fitness penalty exhibited by TSR plants under competitive conditions can be exploited in efforts to overcome herbicide resistance with non-herbicidal techniques. The development of integrated weed management practices that increase competition such as the use of vigorous cultivars, shading crops and controlled mineral deficiency could help control resistant weeds and contribute to further reductions in their seed production.

## Author Contributions

EF, MM, BR, and ZP designed the experiments. EF and MM conducted the experiment. EF, MM, BR, and ZP analyzed data and wrote the paper. All authors read and approved the manuscript.

## Conflict of Interest Statement

The authors declare that the research was conducted in the absence of any commercial or financial relationships that could be construed as a potential conflict of interest.
