# Supplementary material for: Effects of Environmental Conditions on the Fitness Penalty in Herbicide Resistant Brachypodium hybridum
Source: Front Plant Sci. 2017 Feb 3;8:94. doi: 10.3389/fpls.2017.00094 (PMC5289963; doi:10.3389/fpls.2017.00094)
Supplement: Supplementary file 1 [file Presentation_1.PDF]

Supplementary Material

Effect of environmental conditions on the fitness penalty in *Brachypodium hybridum*

Eyal Frenkel, Maor Matzrafi, Baruch Rubin, Zvi Peleg

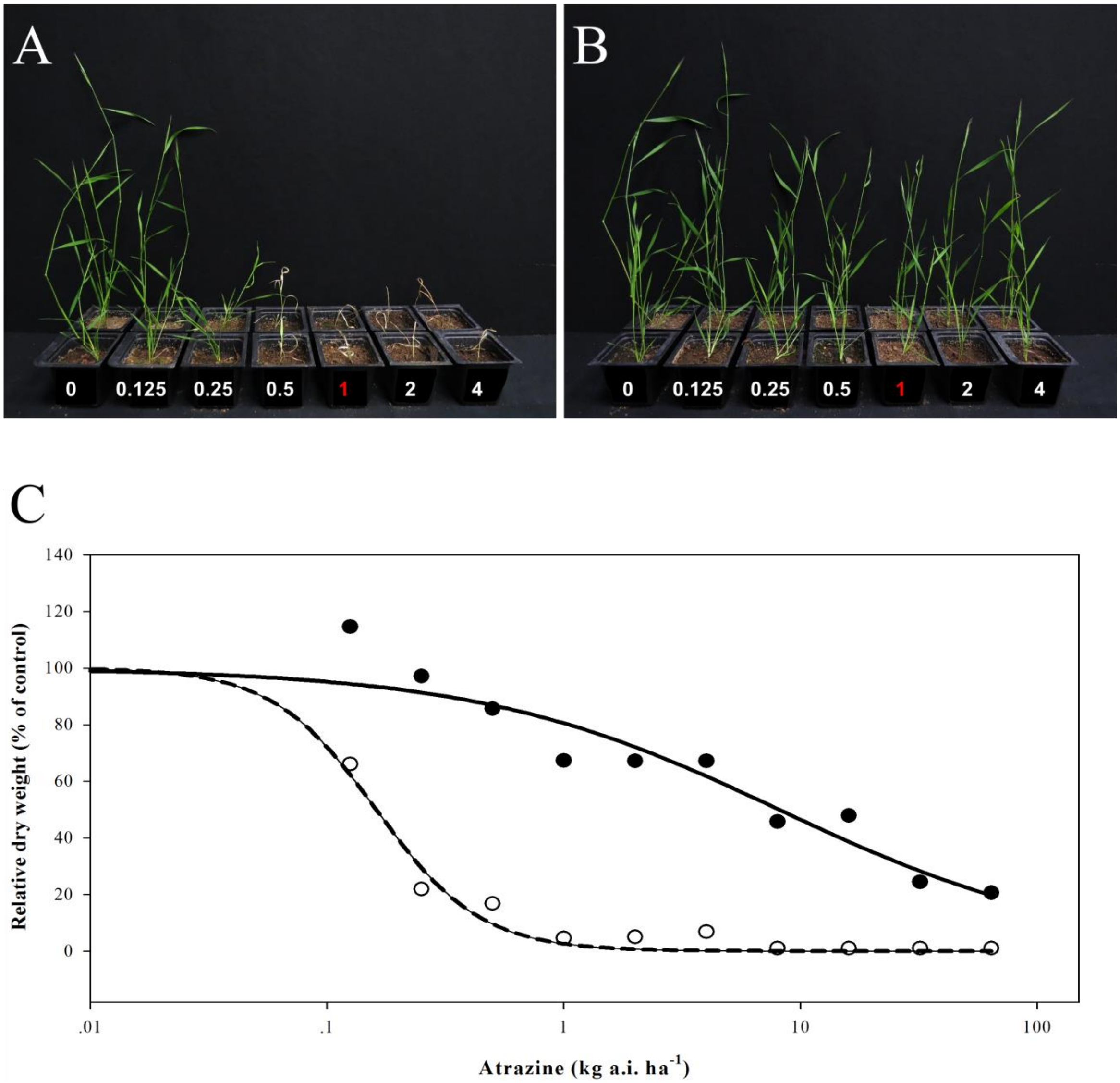

Figure S1. Effect of atrazine applied post-emergence on the shoot dry weight of S (broken) and R (full) B. hybridum accessions. The recommended rate is highlighted in red (A-B). Their ED_{50} values, calculated from the response curves, were 0.161kg ha-1 and 8.124kg ha-1, respectively (C).

Table S1. Composition of nitrogen-rich (100%) and -poor (6%) nutrient solutions.

Chemical compound	Nitrogen rich solution, mM	Nitrogen poor solution, mM
KNO_3	3	-
NH_4NO_3	2	-
$Ca(NO_3)_2$	1	-
$MgSO_4$	1	1
K_2HPO_4	-	0.5
$NaNO_3$	3	1
KCl	-	2
$CaCl_2$	3	4
NaH_2PO_4	0.5	-
EDFS	0.03	0.03
KNO_3	0.001	0.001
$CuSO_4$	0.001	0.001
H_2MoO_4	0.002	0.002
$MnSO_4$	0.050	0.050
H_3BO_3	0.025	0.025
$ZnSO_4$	0.001	0.001

Table S2. Herbicide response parameters of the sensitive (BrI-638) and resistance (R, BrI-637) *Brachypodium hybridum* accessions.

Accession	ED_{50}, kg ha^{-1}	RI
BrI-638	0.161	1
BrI-637	8.124	50.4

Table S3. Effect of day length on heading date of the sensitive (BrI-638) and resistance (BrI-637) *Brachypodium hybridum* accessions.

Accession	Day length	Days to heading
BrI-638	Short	85±0 ***
BrI-637		92±0
BrI-638	Long	39.6±0.93 ***
BrI-637		48±0

*** indicate significant difference between accessions as determined by student-t test at P<0.001.

Table S4. Chlorophyll content and ratio of the sensitive (BrI-638) and resistance (BrI-637) *Brachypodium hybridum* accessions.

Accession	Chl *a, mg/g*	Chl *b, mg/g*	Total Chl, mg/g	Chl *a/b* ratio
BrI-638	2.52±0.09 ***	0.78±0.03 ***	3.29±0.11 ***	3.26±0.05
BrI-637	1.74±0.06	0.53±0.02	2.27±0.09	3.34±0.06

*** indicate significant difference between accessions as determined by student-t test at P<0.001.

Table S5. Grain characteristics of the sensitive (BrI-638) and resistance (BrI-637) *Brachypodium hybridum* accessions.

Accession	Grain weight, mg	Grain area, mm^2	Grain length, mm	Grain width, mm
BrI-638	4.01±0.04***	6.96±0.20***	7.07±0.11**	1.26±0.03**
BrI-637	3.54±0.07	5.96±0.21	6.67±0.11	1.15±0.03

Data represent means and standard errors. ** and *** indicates significant difference between two accessions in each treatment at P<0.01 and P<0.001, respectively.

Table S6. Effect of intra- and inter- species competition between sensitive (S, BrI-638) and resistance (R, BrI-637) *Brachypodium hybridum* accessions and bread wheat (W, *cv*. Zahir) on plants phenology and productivity.

Accession	Competitor	Biomass, g	Fitness penalty[1]	Height, cm	Fitness penalty	# Tillers	Fitness penalty
S	S	0.49±0.01	-33%	49.19±1.49	-9%	6.02±0.28	-51%
S	W	0.33±0.01	***	44.56±1.35	*	2.94±0.07	***
R	R	0.43±0.05	-55%	41.30±0.84	-25%	5.69±0.19	-56%
R	W	0.19±0.01	**	30.67±0.77	***	2.46±0.06	**
W	S	1.80±0.17	8%	35.74±2.71	-4%	-	
W	R	1.97±0.04	n.s.	34.07±1.29	n.s.	-	

[1.] Fitness penalty indicate the relative performance between the S and R accession.

*, **, *** and n.s. indicate significant level of $P \leq 0.05$, $P < 0.01$, $P < 0.001$, and non-significant, respectively.
